# Regulation of factor V by the anticoagulant protease activated protein C: Influence of the B-domain and TFPIα

**DOI:** 10.1016/j.jbc.2022.102558

**Published:** 2022-09-30

**Authors:** Francis Ayombil, Teodolinda Petrillo, Haein Kim, Rodney M. Camire

**Affiliations:** 1 Division of Hematology and the Raymond G. Perelman Center for Cellular and Molecular Therapeutics, The Children’s Hospital of Philadelphia, Philadelphia, Pennsylvania, USA; 2 Department of Pediatrics, Perelman School of Medicine, University of Pennsylvania, Philadelphia, Pennsylvania, USA

**Keywords:** factor V, acidic and basic residues, procofactor, tissue factor pathway inhibitor, factor Va, cofactor, prothrombinase, coagulation factor, thrombin, protein complex, hemostasis, BR, basic region, FV, factor V, FVa, factor Va, FXa, factor Xa, HC, heavy chain, PC, L-α-phosphatidylcholine, PD-FV, plasma-derived FV, PS, L-α-phosphatidylserine, TGA, thrombin generation assay

## Abstract

Activated protein C (APC) is an important anticoagulant protein that regulates thrombin generation through inactivation of factor V (FV) and activated factor V (FVa). The rate of APC inactivation of FV is slower compared to FVa, although proteolysis occurs at the same sites (Arg^306^, Arg^506^, and Arg^679^). The molecular basis for FV resistance to APC is unknown. Further, there is no information about how FV-short, a physiologically relevant isoform of FV with a shortened B-domain, is regulated by APC. Here, we identify the molecular determinants which differentially regulate APC recognition of FV *versus* FVa and uncover how FV-short can be protected from this anticoagulant pathway. Using recombinant FV derivatives and B-domain fragments, we show that the conserved basic region (BR; 963–1008) within the central portion of the B-domain plays a major role in limiting APC cleavage at Arg^506^. Derivatives of FV lacking the BR, including FV-short, were subject to rapid cleavage at Arg^506^ and were inactivated like FVa. The addition of a FV-BR fragment reversed this effect and delayed APC inactivation. We also found that anticoagulant glycoprotein TFPIα, which has a C-terminal BR homologous to FV-BR, protects FV-short from APC inactivation by delaying cleavage at Arg^506^. We conclude that the FV-BR plays a major role in protecting FV from APC inactivation. Using a similar mechanistic strategy, TFPIα also shields FV-short from APC. These findings clarify the resistance of FV to APC, advance our understanding of FV/FVa regulation, and establish a mechanistic framework for manipulating this reaction to alter coagulation.

Coagulation factor V (FV) circulates in blood as an inactive procofactor. It is synthesized as a large multidomain (A1-A2-B-A3-C1-C2) protein in the liver and is also found in the alpha-granules of platelets (1). FV is activated to factor Va (FVa) following proteolytic removal of a large central B-domain (residues 710–1545). Factor Va is a cofactor in the prothrombinase complex and binds factor Xa (FXa) and anionic membranes to facilitate the conversion of prothrombin to thrombin (1, 2). It enhances the relative rate of thrombin generation by five orders of magnitude compared to FXa alone underscoring its critical role in hemostasis (3). Since FV has no procoagulant activity, thrombin or FXa-mediated activation of FV represents a key regulatory point in coagulation.

Mechanistic insights into how the B-domain keeps FV inactive have been made and the steps required to convert it to a procoagulant cofactor have been uncovered (4, 5, 6, 7, 8, 9). The data support a model in which two evolutionary conserved regions found in the B-domain (basic region [BR; 963–1008] and acidic region 2 [AR2; 1493–1537]) work together to block the FXa-binding site on the heavy and light chains (6, 7). Together, the BR-AR2 ensemble enforce the FV procofactor state and effectively prevent the expression of cofactor activity (4, 5, 6, 9). Removing either of these functional landmarks through proteolysis or deletion allows for FXa binding and produces a procoagulant cofactor that can assemble in prothrombinase (4, 5). Importantly, recombinant FV-BR fragments bind with high affinity to physiologic forms of FV that retain AR2 but lack BR. These forms include partially cleaved FV, platelet-derived FV, and a newly identified spliced isoform called FV-short. FV-short was discovered in a family with a moderately severe bleeding disorder (FV-East Texas bleeding disorder). The mutation in the *F5* gene (A2440G; S756G) activates a weak splice site in exon 13 resulting in an abundant, alternatively spliced transcript, which encodes for FV-short (10, 11). Additional families and mutations have been found that yield high levels of FV-short (12, 13, 14). This new form of FV lacks 702 amino acids (Δ756–1458) in the B-domain including the BR but it retains AR2 (Fig. 1*A*). Family members with the mutation have high plasma levels of FV-short (∼2–5 nM) and have elevated TFPIα (10-fold) which form a tight complex in plasma (10, 12). In healthy individuals, splicing occurs at a low level, as FV-short is present in normal plasma at subnanomolar concentration (10). We have recently shown that while FV-short is constitutively active, it binds TFPIα with high affinity, which blocks procoagulant function. However, cleavage at Arg^1545^ relieves this inhibition. These findings show that a key aspect of the regulation of FV is the disengagement of the BR and AR2 and the use of these surfaces by TFPIα to regulate procoagulant function and the initiation of coagulation (8).

**Figure 1:**
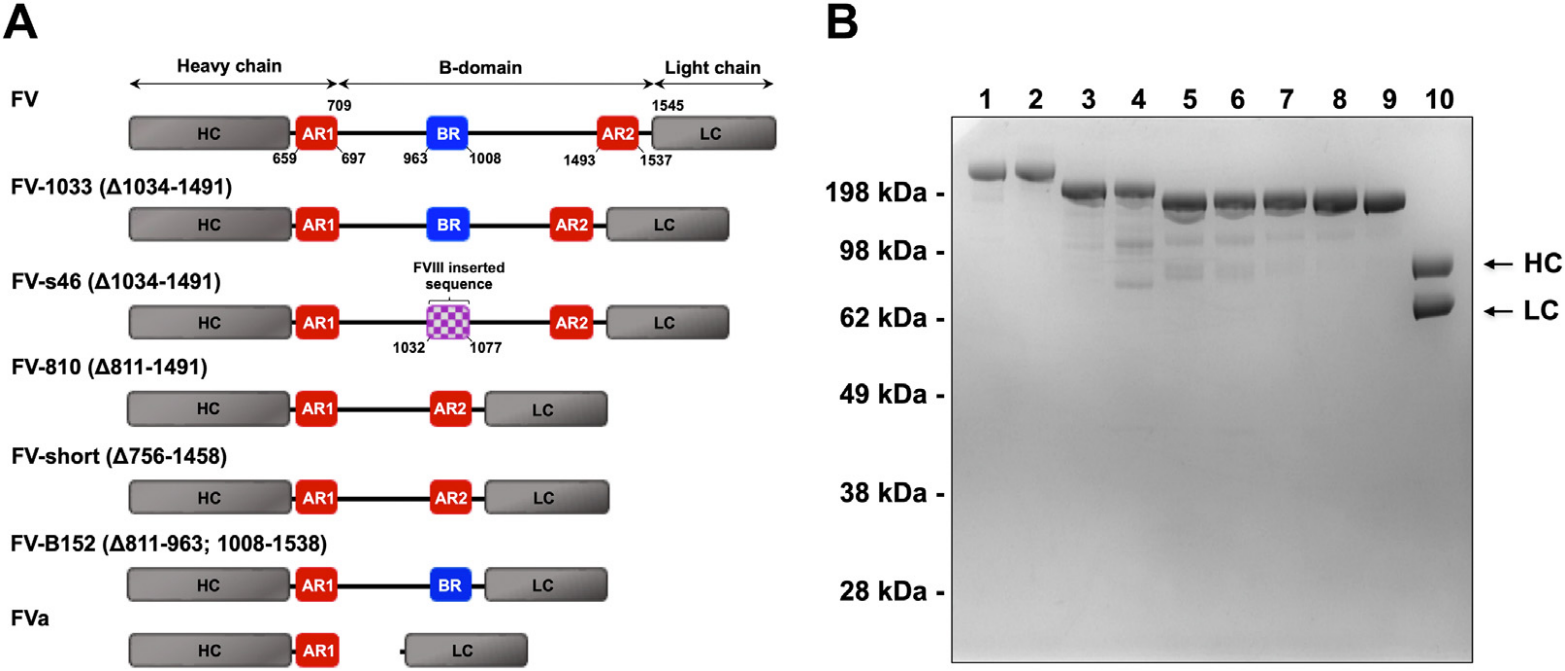
**Schematic representation and SDS-PAGE of purified FV species.***A*, schematic representation of FV shows the HC, LC, and intervening B-domain (residues 710–1545) with the conserved BR (shaded *blue*), AR1, and AR2 (both shaded *red*). FV-1033 and FV-s46 have residues 1034 to 1491 truncated and FV-s46 has BR residues 963 to 1008 replaced with nonhomologous FVIII B-domain sequences (1032–1077; shaded *purple*). FV-810 and FV-short are truncated at residues 811 to 1491 and 756 to 1458, respectively, and are missing the conserved BR. FV-B152 is truncated at residues 811 to 963 and 1008 to 1538 and is missing AR2 but retains the BR. FVa is truncated at 710 to 1545 and is lacking BR and AR2. *B*, protein purity was assessed by SDS-PAGE under reducing conditions and stained with Coomassie Blue R-250. Lanes are: 1, plasma-derived FV, PD-FV; 2, recombinant FV, rFV; 3, FV-1033; 4, FV-s46; 5, FV-810; 6, FV-810-R506Q; 7, FV-810-R306Q; 8, FV-810-QQ; 9, FV-short; and 10, rFVa. FV-B152 was not included on the gel. The apparent molecular weights of protein standards and FVa HC and LC are indicated. BR, basic region; FV, factor V; FVa, factor Va; HC, heavy chain; LC, light chain.

In addition to FV activation and engagement with TFPIα, another point of FV/FV(a) regulation is through the protein C anticoagulant pathway. Protein C is converted to activated protein C (APC) by the thrombin–thrombomodulin complex (15, 16). APC, whose activity is enhanced by its cofactor protein S (PS), regulates hemostasis through the inactivation of the procoagulant activities of FVIIIa and FVa (17, 18, 19, 20, 21). APC cleaves FVa at Arg^306^, Arg^506^, and Arg^679^ within the heavy chain (HC) region. This leads to A2-domain dissociation and loss of cofactor function (22, 23). Studies of FVa inactivation show that cleavage at Arg^506^ is kinetically favored and results in partial inactivation of the cofactor while cleavage at Arg^306^ leads to full inactivation (21, 22, 23, 24). The importance of the Arg^506^ cleavage site is highlighted by the FV-Leiden mutation (Arg^506^ to Gln), which leads to APC resistance and a prothrombotic phenotype (25, 26, 27).

While FVa inactivation by APC has been extensively investigated (20, 25, 28, 29, 30, 31, 32, 33), the physiologic importance of APC cleavage of the procofactor (FV) is less clear. One postulated role relates to the anticoagulant function of FV. FV is thought to act as an anticoagulant cofactor, along with PS, for APC in the inactivation of FVIIIa (34). For FV to exert this anticoagulant effect, it is thought it must be cleaved at Arg^506^, as FV-Leiden does not have anticoagulant function (35, 36). Unlike FVa, FV is cleaved by APC at either Arg^306^ or Arg^506^, with a preference for initial cleavage at Arg^306^ (23, 37). This difference in substrate recognition of FV *versus* FVa contributes to a ∼10-fold reduced rate of inactivation for FV (23).

Despite the longstanding interest in FV/FVa inactivation, the molecular basis for this difference in rate of proteolytic inactivation and APC specificity remains unclear. It is speculated that exposed electropositive surface loops on APC coupled with extended exosite surfaces adjacent to the Arg^506^ cleavage site could explain APC’s differential recognition and the rapid cleavage of FVa relative to FV (38, 39). Additionally, recent cryo-EM data show that the Arg^306^ and Arg^506^ sites appear buried in FV (40) supporting the hypothesis that the bulky B-domain could impair APC recognition *via* unknown steric and/or allosteric determinants.

Given the critical role that FV-BR and AR2 play in the maturation of FVa cofactor function, here we examined whether these B-domain sequences influence APC recognition. Since the FV-BR (and TFPIα-BR) block FXa binding and APC and FXa are known to share a common exosite on FVa, it is reasonable to speculate that FV-BR could alter APC recognition (19, 24, 28, 41). We also studied APC inactivation of FV-short and examined whether TFPIα has any influence on the reaction. Our findings show that both FV-BR and TFPIα-BR have a major influence on APC inactivation of FV and FV-short. When the BR is present in FV or when TFPIα is bound to FV-short, the rate of APC inactivation is delayed significantly with a major effect on the Arg^506^ cleavage site. The studies provide new mechanistic insight into why FV and FVa are differentially regulated by APC and uncover how FV-short is protected from this anticoagulant pathway.

## Results

### Protein preparation

A schematic representation of FV species used in this study is shown in Figure 1*A*. Proteins were recombinantly expressed in baby hamster kidney cells and purified from conditioned media or purified from plasma (plasma-derived FV, PD-FV). FV-1033 is a procofactor-like variant with a truncated B-domain (△1034–1491) that retains the conserved BR and AR2 while FV-s46 is like FV-1033 except its BR is exchanged with 46 amino acids of nonconserved FVIII B-domain sequence (5). FV-810 (△811–1491) is a previously described B-domainless form of FV that lacks the BR and is like the physiologic isoform, FV-short (△756–1458). APC resistance variants of FV-810 with Gln mutations at Arg^306^ (FV-810-R306Q), Arg^506^ (FV-810-R506Q), or both sites (FV-810-QQ) were expressed and purified. FV-B152 is a cofactor-like variant with truncated B-domain regions (△811–963; △1008–1538) and has been previously characterized (6). FV-B152 lacks AR2 but retains the BR. Proteins migrated at the expected position on reduced SDS-PAGE (Fig. 1*B*).

### FV and FVa are differentially proteolyzed by APC

**Figure 2:**
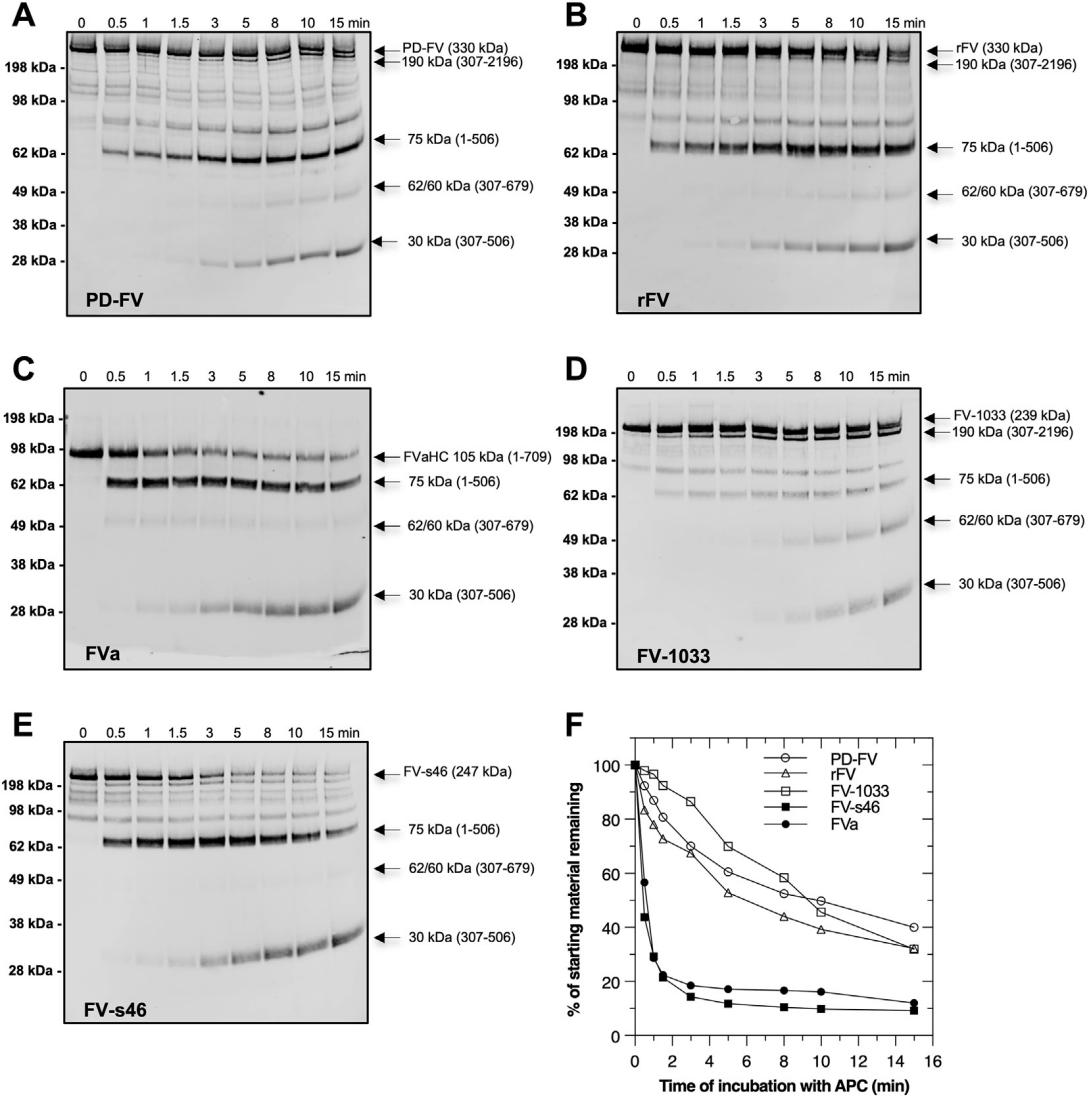
**APC-catalyzed proteolysis of FV and FV species.** Proteolysis of 20 nM PD-FV (*A*), rFV (*B*), FVa (*C*), FV-1033 (*D*), and FV-s46 (*E*) by 1.0 nM APC in reaction mixtures containing 20 μM PCPS in assay buffer was monitored over time (0–15 min, 37 °C). Samples were subjected to SDS-PAGE and cleavage products visualized by immunoblotting using an antihuman FV-HC antibody. Apparent molecular weight markers and key proteolytic fragments are indicated. In (*F*), the intensity of the starting material (uncleaved FV or FV species; *A*–*E*) was plotted as a function of time; PD-FV (○), rFV (Δ), FV-1033 (□), FV-s46 (■), and FVa (●). These data are representative of at least two independent experiments. FV, factor V; FVa, factor Va.

APC inactivates membrane-bound FV/FVa following proteolysis of the HC region at Arg^306^, Arg^506^, and Arg^679^ (23, 28). Consistent with prior findings, the rate of APC inactivation of PD-FV and rFV is slower compared to FVa (Fig. 2, *A–C* and *F*). Western blotting of the HC region shows that FVa is initially cleaved at Arg^506^ (appearance of 75 kDa fragment) while PD-FV and rFV are initially cleaved at either Arg^306^ (appearance of 190 kDa fragment) or Arg^506^ (appearance of 75 kDa fragment) (Fig. 2, *A–C*). These data show that the procofactor has reduced susceptibility to APC-mediated inactivation and is recognized differently compared to FVa under similar experimental conditions. These differences suggest that the B-domain plays a role in altering substrate recognition by APC.

The BR-AR2 (Fig. 1*A*) region within the FV B-domain is necessary and sufficient to keep FV as an inactive procofactor (4, 5, 6, 9). To test whether this region of FV is involved in altering APC recognition, we first prepared two FV derivatives, one with and one without the BR. FV-1033, a procofactor-like variant containing the entire AR1-BR-AR2 region, was inactivated by APC like FV (Fig. 2, *D* and *F*). In contrast, swapping out the BR region with nonconserved B-domain sequences from FVIII (FV-s46) (5) significantly enhanced the rate of APC inactivation and shifted the initial cleavage to Arg^506^, like FVa (Fig. 2, *E* and *F*). These data show that the endogenous FV-BR somehow influences APC recognition of the procofactor molecule. However, the BR alone is not sufficient to alter APC recognition. For example, FV-B152, which retains the BR but lacks AR2 (6), was inactivated rapidly by APC like FVa with initial cleavage primarily occurring at Arg^506^ (Fig. S1, *A* and *C*). These data show that for the endogenous FV-BR to alter APC recognition, it must involve an interaction with AR2.

### The FV-BR directly influences APC recognition

**Figure 3:**
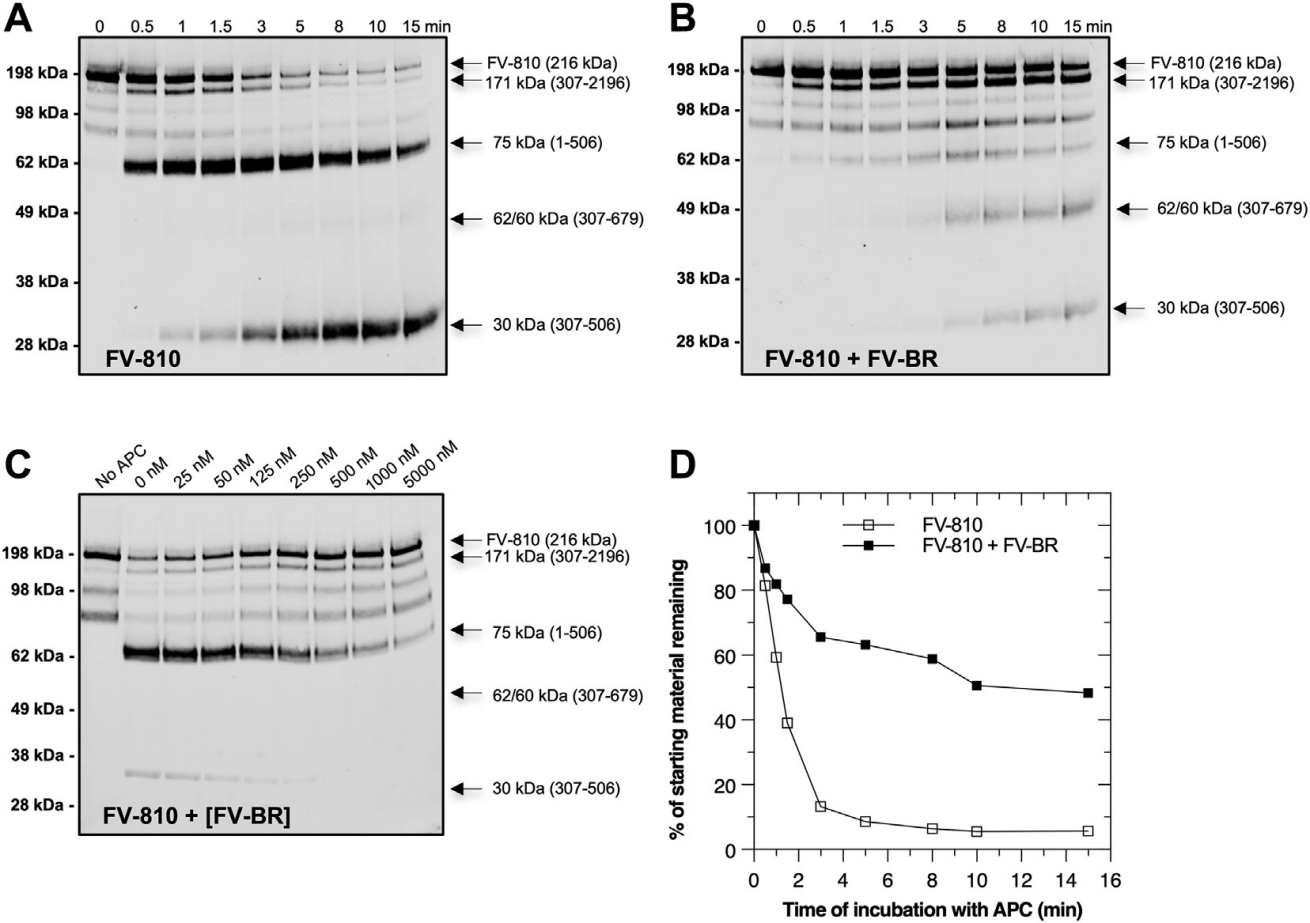
**Proteolysis of FV-810 is impaired when bound to FV-BR.** Cleavage of 20 nM FV-810 by 1.0 nM APC in the absence (*A*) or presence of FV-BR at a fixed concentration (250 nM; *B*) over time or increasing FV-BR concentration (0–5 μM; *C*) at a single end point (3 min) was monitored in assay buffer. Samples were resolved by SDS-PAGE and immunoblotted as in Figure 2. In panel (*D*), band density of starting material (*A* & *B*) was expressed as a function of time; FV-810 (□) and FV-810 + FV-BR (■). Data are representative of two or more similar experiments. BR, basic region; FV, factor V.

To examine these ideas further, we investigated the inactivation of FV-810 by APC in the presence and absence of exogenous FV-BR fragment. FV-810 (Fig. 1*A*) retains AR2 and binds the FV-BR fragment with high affinity (*K*_*D*_ ∼ 1–2 nM) (7). In the absence of the exogeneous FV-BR fragment, FV-810 was inactivated by APC like FVa where the HC region was rapidly cleaved at Arg^506^ with the appearance of 75 kDa fragment (Fig. 3, *A* and *D*). The accumulation of the 30 kDa fragment (*via* subsequent cleavage at Arg^306^) was visible over time consistent with inactivation of FV-810. In contrast to these data, when excess FV-BR was added in the reaction mixture, the rate of APC inactivation of FV-810 was impaired and initial cleavage in the HC region now favored Arg^306^ (indicated by accumulation of 171 kDa fragment) *versus* Arg^506^ (Fig. 3, *B* and *D*). The FV-BR fragment had no influence on the rate of FV inactivation by APC since FV has its own internal BR. Further, FV-BR had no effect on FVa inactivation since FV-BR cannot bind FVa (Fig. S2 and Table 1) and it had no effect on FV-B152, which lacks AR2 (Fig. S1, *B* and *C*). In control experiments, we were not able to detect binding between the BR fragment and APC by fluorescence methods and it had no influence on APC proteolytic activity using a peptidyl substrate (Table 1 and Fig. S3). This suggests that the FV-BR fragment does not interact with or alter the function of APC directly. Additional experiments showed that increasing concentrations of FV-BR reduced APC inactivation of FV-810 (Fig. 3*C*). These data show that the FV-BR when bound to AR2 protects FV from inactivation and appears to have an influence at/near the Arg^506^ region either directly or allosterically.

**Table 1:** Binding constants for TFPIα-BR

FV species	K_d_ ± S.D.	n ± S.D.
	nM	mol/mol
FV-s46	2.17 ± 0.25	1.78 ± 0.04
FV-short	1.56 ± 0.23	1.18 ± 0.03
FV-810	1.78 ± 0.28	1.29 ± 0.04
FV-810-R306Q	2.15 ± 0.34	1.44 ± 0.05
FV-810-R506Q	1.72 ± 0.29	1.43 ± 0.04
FV-810-QQ	2.46 ± 0.36	1.18 ± 0.04
FVa	NA	NA
FV-1033	NA	NA
APC_i_	NA	NA

Fluorescence data were obtained by titrating FV species or APC_i_ into reaction mixtures containing 25 nM OG_488_-TFPIα-BR and 50 μM PCPS vesicles in assay buffer as described under ‘‘Experimental Procedures.’’ The data were fit to a model for tight binding to obtain the binding constant (*K*_*D*_) and stoichiometry (*n*). NA indicates that there was no detectable binding at the concentrations evaluated. Data represent an average of two to three independent experiments.

**Figure 4:**
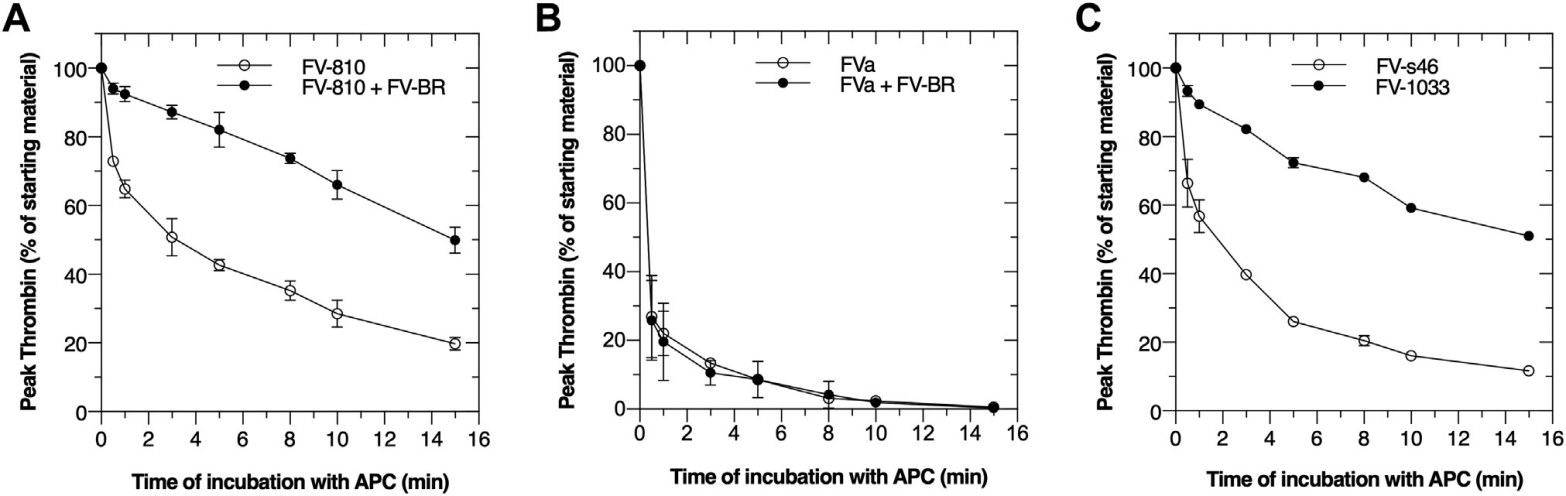
**FV-BR impacts susceptibility to APC proteolysis and loss of cofactor activity.** APC (1.0 nM) inactivation of 20 nM FV-810 (*A*) or FVa (*B*) was assessed in the absence (○) or presence (●) of 250 nM FV-BR in assay buffer over time. In panel (*C*), FV-1033 and FV-s46 inactivation by APC was followed in the absence of exogeneous FV-BR. Residual cofactor activity was assessed in a calcium-initiated TGA using FV-DP containing 2 pM TF/4 μM phospholipid and fluorogenic thrombin substrate (Z-GGR-AMC). Data are shown as percentage peak and are an average of three similar experiments (mean ± SD)%. BR, basic region; FV, factor V; TGA, thrombin generation assay.

The finding that FV-BR protects FV-810 from APC cleavage was further evaluated using functional measurements as assessed in a tissue factor initiated thrombin generation assay (TGA). As shown in Figure 4*A*, when FV-BR was bound to FV-810, APC was much less effective at reducing thrombin generation as assessed by peak thrombin remaining. Since FV-BR cannot bind FVa due to the absence of AR2 (Fig. 1*A*), FV-BR had no effect on APC inactivation of FVa (Fig. 4*B*). Consistent with these data, the rate of APC inactivation of FV-1033 compared to FV-s46 as assessed by TGA was much slower when the internal BR region was present (Fig. 4*C*).

### FV-short–TFPIα complex is resistant to APC inactivation

FV-short is a physiologically relevant spliced isoform of FV and is structurally like FV-810 (Fig. 1*A*). It forms a high affinity complex with TFPIα *via* its C-terminal BR, which dampens cofactor function by blocking FXa binding and hence prothrombinase assembly (10, 13, 42).

**Figure 5:**
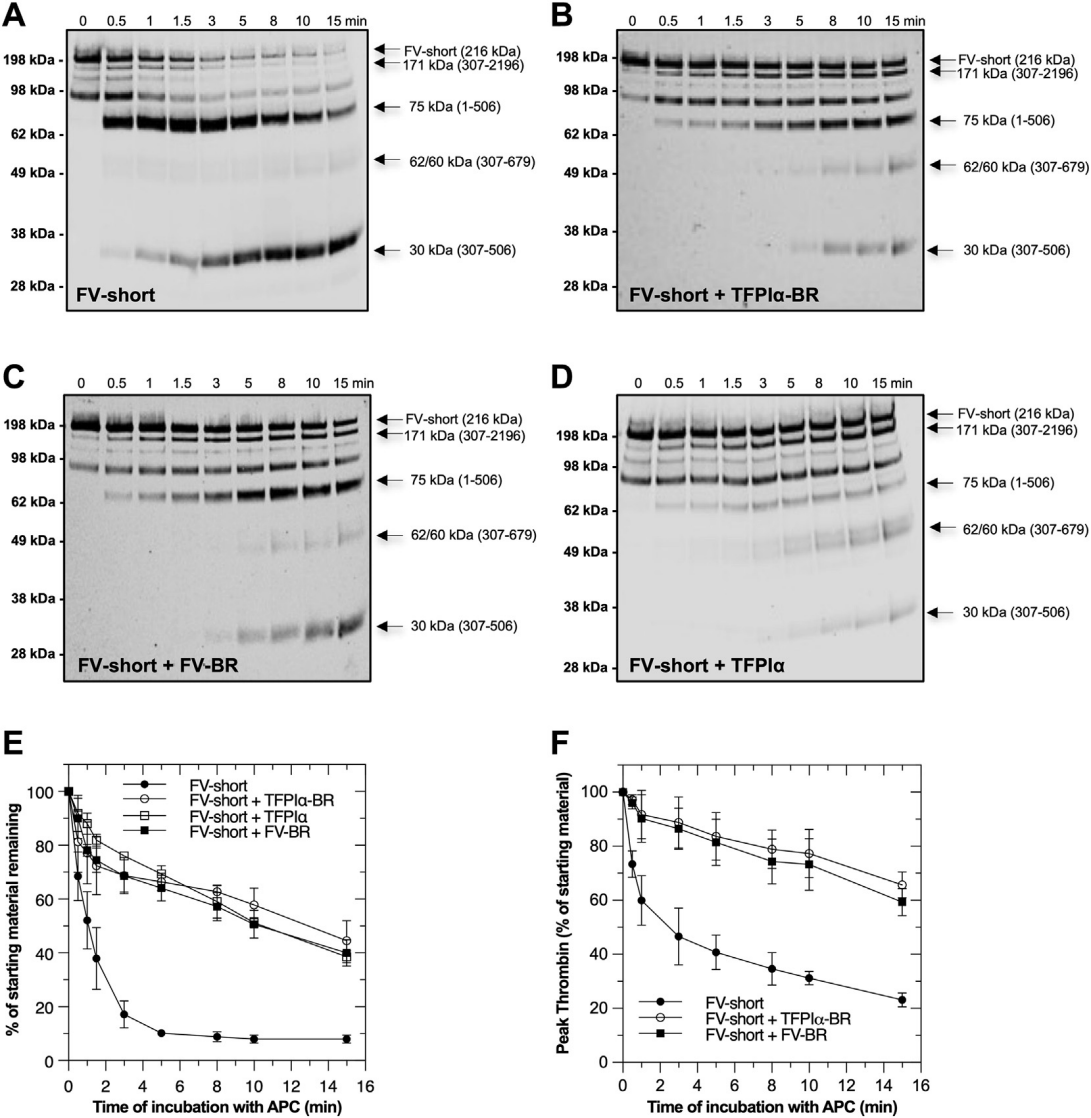
**TFPIα, TFPIα-BR, and FV-BR impair FV-short proteolysis by APC.** Immunoblots of FV-short (20 nM) proteolysis by APC (1.0 nM) in the absence (*A*) or presence of 250 nM TFPIα-BR (*B*), FV-BR (*C*), or TFPIα (*D*) are indicated. Panel (*E*) shows quantitative densitometry data of panels (*A–D*) expressed as a percentage of starting material remaining over time for FV-short (●) with TFPIα-BR (○), TFPIα (□), or FV-BR (■). Panel (*F*) is data from TGA functional assay expressed as peak thrombin. Plotted data are an average of three similar experiments (mean ± SD)%. BR, basic region; FV, factor V.

We next examined whether TFPIα *via* its C-terminal BR, protects FV-short from APC inactivation. Like FV-810, FV-short was rapidly cleaved in the HC region by APC initially at Arg^506^ (75 kDa fragment), then at Arg^306^ (30 kDa fragment) (Fig. 5, *A* and *E*). The addition of exogenous TFPIα-BR fragment noticeably slowed the rate of APC inactivation and altered the initial cleavage from Arg^506^ to Arg^306^ (Fig. 5, *B* and *E*). As expected, similar results were obtained with FV-BR (Fig. 5*C*) as the fragments are homologous and both bind to FV-short with high affinity. Importantly, saturating amounts of full-length TFPIα also similarly delayed APC inactivation of FV-short, although its impact on cleavage at Arg^506^ appears to be more prominent than TFPIα-BR or FV-BR (Fig. 5, *D* and *E*). Consistent with the Western blotting results, TFPIα-BR protected FV-short from APC inactivation as assessed by functional TGA (Fig. 5*F*). Together, these data show that the FV-short–TFPIα complex is protected from APC inactivation. This resistance to APC is mediated through its C-terminal BR region.

### Impaired APC cleavage at Arg^506^ is mediated by the FV-BR

**Figure 6:**
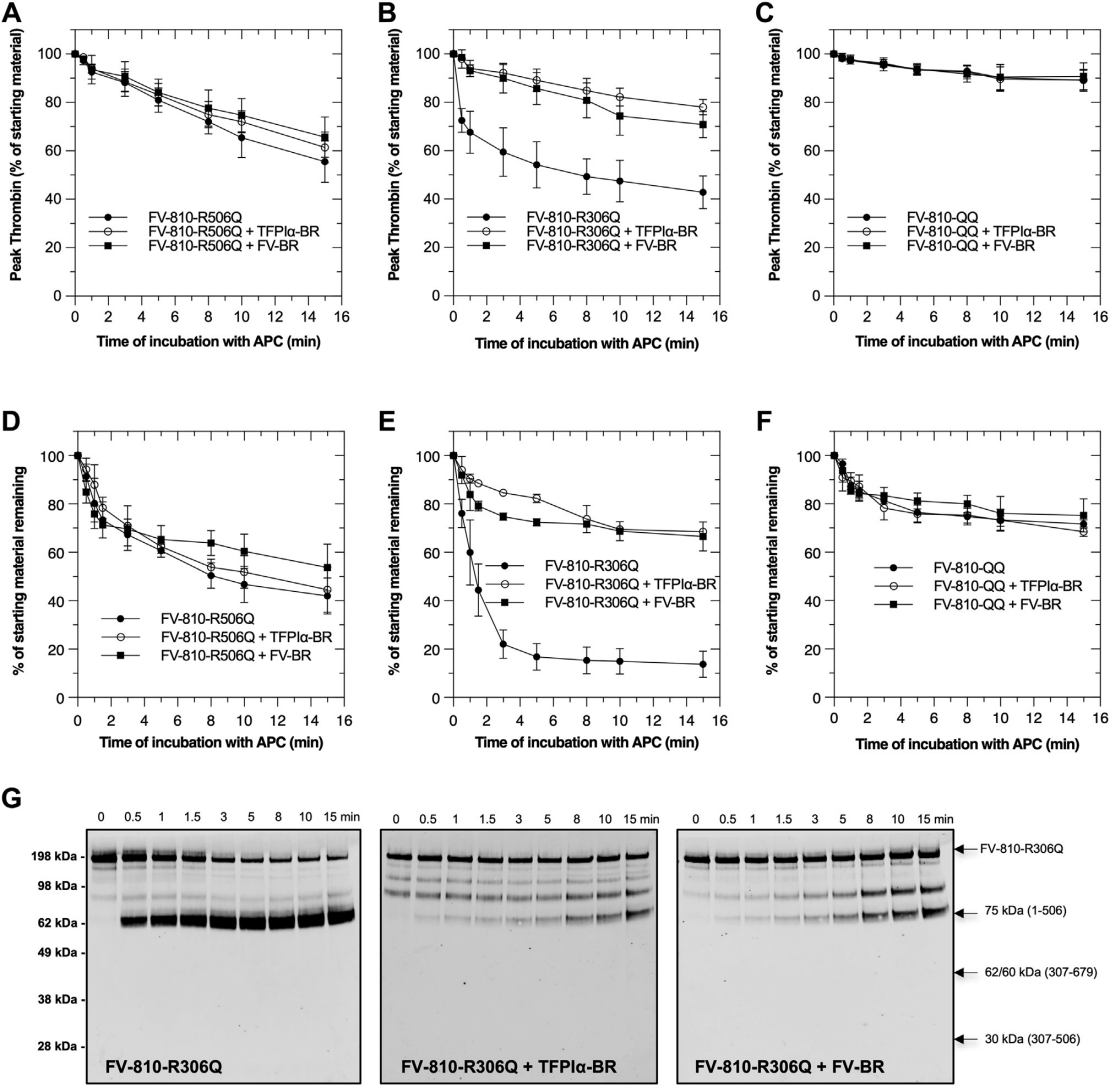
**Cleavage at Arg**^**506**^**is altered by BR fragments.** Proteolysis of APC cleavage site mutants in FV-810 was followed in two assays in the absence (●) and presence of TFPIα-BR (○) or FV-BR (■) as described in ‘‘Experimental Procedures.’’ Data are expressed as percentages of residual cofactor activity (peak thrombin) in TGAs and starting material remaining (densitometry of immunoblots) over time for FV-810-R506Q (*A* & *D*), FV-810-R306Q (*B* & *E*), and FV-810-QQ (*C* & *F*), respectively (n = 3; mean ± SD) %. In panel (*G*), representative immunoblots for the data in panel (*E*) are shown. BR, basic region; FV, factor V; TGA, thrombin generation assay.

Our data suggest that the BR, either within the FV B-domain, added exogenously as a fragment, or derived from the C terminus of TFPIα, protects FV-810 and FV-short from APC and alters access to the Arg^506^ cleavage site. To evaluate this in more detail, we prepared APC cleavage site variants of FV-810 including FV-810-R306Q, FV-810-R506Q, and FV-810-R306Q/R506Q; FV-810-QQ. Each of these FV-810 derivatives bound BR fragments with affinities like FV-810 and FV-short (Table 1). APC inactivation of each variant was assessed by Western blotting and TGA in the absence or presence of FV-BR or TFPIα-BR (Fig. 6 and Table 2). When APC cleavage of FV-810 is isolated to Arg^306^ (using the FV-810-R506Q mutant), FV-BR or TFPIα-BR had no impact on the rate of inactivation (Fig. 6, *A* and *D*). In contrast, when APC cleavage is isolated to Arg^506^ (using the FV-810-R306Q mutant), FV-BR or TFPIα-BR had a major impact on APC cleavage and loss of cofactor activity protecting the protein from APC-mediated proteolytic inactivation *via* impaired appearance of the 75 kDa fragment (Fig. 6, *B*, *E* and *G*). FV-BR or TFPIα-BR had no impact on FV-QQ since this variant is already APC resistant (Fig. 6, *C* and *F*). We speculate that the ∼20% loss in FV-810-QQ cofactor function is due to APC cleavage at Arg^679^, a minor cleavage site for APC (23). Together, these data show that the BR derived from FV or TFPIα delays the rate of APC inactivation largely by altering access to the Arg^506^ cleavage site. This could occur in a direct way through steric effects or the cleavage site could be altered allosterically when BR is engaged with AR2 in FV/FV-short.

**Table 2:** Rate constant of FV inactivation by APC with or without BR fragments

FV species	No BR	+TFPIα-BR	+FV-BR	Reduced fold change of inactivation	
	kobs ± S.D.	kobs ± S.D.	k_obs_ ± S.D.		
	min^−1^	min^−1^	min^−1^	+TFPIα-BR	+FV-BR
FV-s46	1.25 ± 0.19	0.15 ± 0.02	0.23 ± 0.02	8.3	5.4
FV-short	0.71 ± 0.14	0.05 ± 0.06	0.05 ± 0.05	14.2	15.7
FV-810	0.34 ± 0.06	0.02 ± 0.02	0.02 ± 0.01	17.0	17.0
FV-810-R306Q	1.09 ± 0.28	0.09 ± 0.05	0.09 ± 0.05	12.1	12.1
FV-810-R506Q	0.05 ± 0.03	0.06 ± 0.03	0.06 ± 0.05	0.8	0.8
FV-810-QQ	0.12 ± 0.08	0.13 ± 0.08	0.20 ± 0.13	0.9	0.6
FVa	2.60 ± 0.39	3.09 ± 0.37	2.74 ± 0.42	0.8	0.9
FV-1033	0.04 ± 0.03	0.06 ± 0.03	0.07 ± 0.02	0.7	0.6

The rate constant of FV inactivation in the presence or absence of BR fragments was determined by plotting % peak thrombin as a function of time and the data were fit to a one phase decay equation (Graphpad Prism v9). Fold change of inactivation is calculated by dividing the rate, *k*_*obs*_ (no BR) by *k*_*obs*_ (+BR). Data represent an average of three independent experiments.

## Discussion

The results of our study provide new insights into the molecular basis by which FV, FV-short, and FVa are differentially recognized by APC and help clarify a longstanding gap in the field. We show that BR sequences, derived from the FV B-domain or from the C terminus of TFPIα, have a major influence on APC substrate recognition. While anchored to AR2, the BR alters APC recognition either directly or indirectly by delaying cleavage at Arg^506^. When the BR is present in FV, the rate of APC inactivation is delayed at least 10-fold compared to FVa or FV derivatives lacking the BR. This mechanism is mimicked by the BR of TFPIα. The protection of FV and FV-short by FV-BR or TFPIα-BR preserve these proteins for eventual procoagulant activity and may also have implications for their anticoagulant function (34, 43).

The FV-BR and AR2 represent functional landmarks that interact with each other and are the minimal B-domain sequences needed to enforce the procofactor state (4, 5, 6). When bound to AR2, FV-BR, or TFPIα-BR, block FXa binding to the heavy/light chain. This explains why the BR inhibits procoagulant function and how FV is maintained as a procofactor (7, 44). We now show here that the FV-BR or TFPIα-BR also have a major impact on the rate of APC inactivation predominately through delayed cleavage at Arg^506^. Replacing the internal FV-BR with nonhomologous B-domain sequences from FVIII accelerated APC inactivation by enhancing cleavage at Arg^506^. These findings of rapid initial cleavage at Arg^506^ mimic what is seen with FVa, FV-810, and FV-short. However, when FV-BR, TFPIα-BR, or full-length TFPIα are bound to FV-810 or FV-short, each complex was resistant to APC with delayed cleavage at Arg^506^. Overall, the data show that cofactor-like species are preferentially cleaved at Arg^506^, whereas cleavage at Arg^306^ is favored in the procofactor. This preference is largely, if not exclusively, driven by FV-BR or TFPIα-BR anchored to AR2.

How can the present results be put into context with available structural information about FV and FVa? Currently there is no X-ray structure of full-length FV and the structure of bovine FVa lacks the A2 domain. A preliminary high resolution structure of FV-810 has been reported; but, resolution of the APC cleavage sites is poor (45). This was also the case with a FV(a) ortholog from *Pseudonaja textilis* (46). In the FV-810 structure, both AR1 and AR2 are in proximity and sit at the outer edge of A2/A3 domain interface providing an extended surface for FV-BR or TFPIα-BR. These sequences are at a distance from Arg^306^ and Arg^506^ in the structure. Further, it was found that FV-BR is structured and makes intimate contacts with both AR1 and AR2. It is reasonable to speculate based on biochemical work that the TFPIα-BR engages AR1 and AR2 in a similar way (47). More recently, Ruben *et al*. using cryo-EM found that Arg^506^ and Arg^306^ are solvent exposed in the cofactor but ∼75% buried in the procofactor (40). This would suggest that removal of the B-domain exposes the APC cleavage sites on the heavy chain. Unfortunately, the B-domain was found to be mostly disordered including the BR and AR2 regions. Thus, while this structure does not implicate a specific part of the B-domain, it is clear this domain influences exposure of the APC cleavage sites. These new structural findings and work reported here provide evidence that the BR from the B-domain contributes in a major way to altering the APC cleavage sites. Future structural studies with FV-short bound to BR fragments could provide important insights into how the Arg^306^ and Arg^506^ cleavage sites are altered.

While our data provide new mechanistic insights, the physiologic significance of our findings with respect to regulation of coagulation balance is not completely clear. Due to complexities of data interpretation and other factors, employing more physiologic experimental systems to interrogate the role of the BR in regulating FV/FV-short inactivation by APC is difficult. While this is a limitation of the current study, our mechanistic findings can be put into some physiologic context with existing studies in the literature. For example, the protection of FV and FV-short from APC by the FV-BR and TFPIα-BR, respectively, could have physiologic consequences beyond preserving procoagulant function. In addition to their procoagulant roles, both FV and FV-short are thought to have anticoagulant function. For FV, through characterization of FV-Leiden, it was found that membrane-bound FV serves as an APC cofactor together with PS in the inactivation of FVIIIa in the assembled FIXa/FVIIIa intrinsic Xase complex (34, 36). FV cleaved at Arg^506^ greatly stimulates the APC cofactor activity and helps explain why FV-Leiden is a poor cofactor for this reaction. However, once FV is converted to FVa, it loses this anticoagulant function. Specifically, it was found that when FV is cleaved at Arg^1545^, APC cofactor activity is abolished and sequences within AR2 (1476–1545) are critical to this anticoagulant function (48). At present, it is not clear how this region contributes to this anticoagulant effect. However, AR2 binds to the BR, suggesting these molecular interactions may influence the FV anticoagulant effect. The potential physiologic impact here with respect to our findings could relate to the FV-BR-AR2 interaction playing a major role in the anticoagulant function of FV.

Another potential physiologic link is related to the role that FV-short may play through its connection with TFPIα. FV-short binds with high affinity to TFPIα and forms a complex with it in plasma, effectively regulating the circulating levels of free TFPIα. Evidence from this comes from multiple reports of individuals with elevated levels of FV-short due to enhanced splicing caused by mutations, which leads to correspondingly high levels of TFPIα (10, 11, 12, 13, 14). Additionally, the inhibition of FXa by TFPIα is stimulated by both PS and FV-short (43). Thus, the protection of FV-short by TFPIα from APC inactivation may play an important role in preserving the optimal anticoagulant function of TFPIα. This pathway is thought to play a key role in limiting FXa formation and the initiation of coagulation; however, it is not clear at this point how important a role FV-short plays in regulating TFPIα activity *in vivo*. It is likely more than just a carrier for TFPIα; but, whether its procoagulant or anticoagulant role contribute to normal hemostasis in a meaningful way remains to be determined. Our work however shows that FV-short is differentially regulated by APC depending on whether it is bound to TFPIα.

One additional connection of our findings to potential physiologic significance relates to the regulation of the platelet FV pool. In addition to what is found in plasma, ∼25% of the total FV pool is in platelets. Platelet-derived FV is a mixture of full-length FV, partially cleaved forms of FV processed within the B-domain, and FVa (42, 49). It has been previously reported that platelet-derived FV/FV(a) is variably resistant to inactivation by APC (50). Subsequent work attributed this APC resistance to reduced/delayed cleavage at Arg^506^ (51); however, the mechanistic basis for the observation was not clear. A plausible explanation is that forms of platelet FV/Va with some B-domain attached to the light chain including AR2 is bound to platelet-derived TFPIα *via* its BR. It is well known that platelets release TFPIα upon activation. While platelet-derived FV is different from FV-short, it is thought to be partially cleaved in the B-domain and retains AR2, which would enable binding to TFPIα (42). Future studies would need to test this hypothesis, but based on the observations of the current study, TFPIα may contribute to the observed APC resistance of platelet-derived FV/FV(a).

In summary, we identified the mechanism by which FV, FV-short, and FVa are differentially recognized by APC. The BR within the FV B-domain plays a major role in influencing APC inactivation of the procofactor by delaying cleavage at Arg^506^. In a similar way, FV-short is also protected from APC inactivation by TFPIα *via* its C-terminal BR. This likely has a net positive procoagulant effect as both FV and FV-short are preserved. However, it is difficult at this point to assess how this impacts the anticoagulant function of these proteins especially for FV-short, which acts as a TFPIα cofactor for FXa inhibition (43). Uncovering this mechanism has implications for understanding how FV and FV-short are regulated and potentially provide a pathway to modulate the function of these proteins.

## Experimental procedures

### Reagents

Oregon Green 488 maleimide (OG_488_) and succinimidyl acetothioacetate were obtained from Life Technology. FV affinity purification resin (AHV-5101-Seph), mAbs against the HC of human FV (AHV #5146), and Phe-Pro-Arg-chloromethylketone (FPRck) were purchased from Hematologic Technologies. L-α-phosphatidylcholine, PC, (egg, chicken) and L-α-phosphatidylserine, PS, (brain, porcine) were from Avanti Polar Lipids Inc. PCPS phospholipid vesicles comprised of 75% PC (w/w) and 25% PS (w/w) were prepared and characterized as previously described (52). Tissue culture reagents were purchased from Invitrogen and Sigma. Proprietary Western blocking reagent and insulin-transferrin-sodium selenite were obtained from Roche Applied Science. Rabbit antimouse IgG antibodies conjugated to IRDyLight 800 were purchased from Rockland Inc. Fluorogenic thrombin substrate Z-Gly-Gly-Arg-AMC reconstituted in 15 mM CaCl_2_ was obtained from Bachem Bioscience Inc. Technothrombin reagent RB was purchased from Diapharma Group Inc. FV-deficient plasma (FV-DP) was obtained from George King Bio-medical Inc.

### Proteins

Human APC and active site blocked APC (FPR-APC; APCi) were purchased from Hematologic Technologies and Enzyme Research Laboratories, respectively. Plasma-derived FV was purified from plasma donated by the plasmapheresis unit of the University of Pennsylvania using both affinity and ion exchange chromatography protocols as previously described (53, 54). Recombinant FV, FV-1033, FV-s46, FV-short, FV-810, and its Arg to Gln APC resistant variants, FV-810-R306Q, FV-810-R506Q, and FV-810-R306Q/R506Q (FV-810-QQ), were expressed in baby hamster kidney cells and purified by ion exchange chromatography as previously described (4, 5, 9). Human FVa was prepared by thrombin activation of recombinant FV-short or FV-810 and purified as described previously (4, 55). Recombinant tissue factor pathway inhibitor-alpha (TFPIα) was purchased from Biolegend. Recombinant BR fragments derived from the C-terminal region of TFPIα (TFPIα-BR, residues 240–265), the FV B-domain region (FV-BR, 951–1008), and their Cys derivatives were expressed in SUMO-pro bacteria (Life-Sensors), purified, and fluorescently labeled with OG_488_ as previously described (7, 42). Protein concentrations were determined using the following molecular weights and extinction coefficients (E_280nm_^0.1%^): FV-short, FV-810, FV-810-R306Q, FV-810-R506Q, and FV-810-QQ, 216,000, 1.54 (4); FVa, 175,000, 1.78 (4); FV-1033, 239,000, 1.52 (5); FV-s46, 247,000, 1.52 (5); PD-FV and rFV, 330,000, 0.96 (55); TFPIα, 42,000, 0.67 (56).

### Proteolysis of FV species by APC

Factor V and various FV species (20 nM) were added to a reaction mixture containing PCPS vesicles (20 μM) in 20 mM Hepes, 150 mM NaCl, 5 mM CaCl_2_, and 0.1% polyethylene glycol-8000 buffer (HBS/Ca^2+^/PEG, pH 7.4; assay buffer). The reaction was preincubated (5 min, 37 °C) with or without FV-BR, TFPIα-BR, or TFPIα (250 nM) followed by the addition of APC (1 nM) to initiate the reaction. Aliquots were removed at various time points and quenched in 4× NuPAGE LDS sample buffer (106 mM Tris HCl, 141 mM Tris base, 2% LDS, 10% glycerol, 0.51 mM EDTA, 0.22 mM SERVA Blue G250, 0.175 mM phenol red, 50 mM DTT; pH 8.5, 1.2× final). Samples were subsequently boiled (6 min, 80 °C) and stored at −80 °C until analyses by immunoblotting.

### Thrombin generation assay

Residual cofactor activity was monitored in a two-stage assay as follows: following APC-mediated proteolysis of FV species (20 nM) with or without BR fragments (250 nM) in a reverse time course (15–0 min) and samples were diluted 40-fold in HBS/PEG, pH 7.4 (without calcium). In the second assay, 0.5 nM proteolyzed FV species (final) at each time point was immediately added to 40 μl of FV-DP in a microtiter plate (F16 black Maxisorp; Nunc). The TGA reaction was initiated by addition of 10 μl Technothrombin RB reagent (containing ∼2 pM tissue factor, TF; 4 μM phospholipids) followed by the fluorogenic substrate Z-Gly-Gly-Arg-AMC in 15 mM CaCl_2_ (50 μl; 0.5 mM final). The fluorescence was monitored with filters set at 360 nm excitation and 460 nm emission (1 min interval, 90 min, 37 °C) using a Spectramax M2^e^ plate reader (Molecular Devices).

### Fluorescence anisotropy assay

Steady-state fluorescence binding was performed in a QuantaMaster spectrophotometer (Photon Technology International) fitted with long-pass filters (KV500, CVI Melles) as previously described (7, 42, 57). In direct binding studies, FV species or APCi was titrated (0–100 nM) into a 1 cm^2^ quartz cuvette containing a 2.5 ml reaction mixture of OG_488_-TFPIα-BR (25 nM) and PCPS vesicles (50 μM) in HBS/Ca^2+/^PEG, pH 7.4. The change in fluorescence anisotropy, Δr, (λ_ex_ = 480 nm; λ_em_ = 520 nm) was followed at 25 °C over time.

### SDS-PAGE and immunoblotting analyses

Proteins were subjected to gel electrophoresis using 4% to 12% gradient or 10% NuPage gels (Invitrogen) under reducing conditions using Mops. Proteins were subsequently transferred onto nitrocellulose membranes using a dry iBlot system (Invitrogen) followed by blocking with Roche proprietary blocking reagent. Membranes were probed with a mouse antihuman FV HC mAb (AHV #5146, primary antibody) followed by rabbit antimouse IgG antibody conjugated to IRDyLight 800 (fluorescently labeled secondary antibody). Proteolytic products were visualized by scanning blots in an Odyssey Infrared Imaging Instrument (Li-cor Biosciences).

### Densitometry and data analysis

To analyze protein band intensities on Western blots by quantitative densitometry, gamma and image settings were adjusted to fall within the desired linear range and band intensities were measured using the Image Studio Lite software (Licor). The data were normalized as described previously (58, 59) and fit to a single exponential curve using GraphPad Prism v.9.0 (GraphPad Software) to obtain the first-order rate constants, *k*_*obs*_ (min^−1^) of substrate consumption. Data obtained from direct fluorescence anisotropy experiments were fit to Win_Lsq8 software (Provided by Dr. Sriram Krishnaswamy, UPENN/CHOP) to determine the equilibrium dissociation constants (*K*_*D*_) and stoichiometry (*n*) of protein binding interactions as described previously (7, 57).

## Data availability

Data for all figures are contained within the manuscript. Additional data backing the kinetic analyses in the tables are available from the corresponding author upon request.

## Supporting information

This article contains supporting information.

## Conflict of interest

The authors declare that they have no conflicts of interest with the contents of this article.

## References

[1] Mann K.G., Nesheim M.E., Church W.R., Haley P.E., Krishnaswamy S. (1990). Surface dependent reactions of the vitamin K-dependent enzyme complexes. Blood.

[2] Camire R.M., Bos M.H. (2009). The molecular basis of factor V and VIII procofactor activation. J. Thromb. Haemost..

[3] Nesheim M.E., Taswell J.B., Mann K.G. (1979). The contribution of bovine factor V and factor Va to the activity of prothrombinase. J. Biol. Chem..

[4] Toso R., Camire R.M. (2004). Removal of B-domain sequences from factor V rather than specific proteolysis underlies the mechanism by which cofactor function is realized. J. Biol. Chem..

[5] Zhu H., Toso R., Camire R.M. (2007). Inhibitory sequences within the B-domain stabilize circulating factor V in an inactive state. J. Biol. Chem..

[6] Mettine H.A.B., Camire M.R. (2012). A Bipartite autoinhibitory region within the B-domain suppresses function in factor V. J. Biol. Chem..

[7] Bunce M.W., Bos M.H.A., Krishnaswamy S., Camire R.M. (2013). Restoring the procofactor state of factor Va-like variants by complementation with B-domain peptides. J. Biol. Chem..

[8] Petrillo T., Ayombil F., van’t Veer C., Camire R.M. (2021). Regulation of factor V and factor V-short by TFPIα: relationship between B-domain proteolysis and binding. J. Biol. Chem..

[9] Kane W.H., Devore-Carter D., Ortel T.L. (1990). Expression and characterization of recombinant human factor V and a mutant lacking a major portion of the connecting region. Biochemistry.

[10] Vincent L.M., Tran S., Livaja R., Bensend T.A., Milewicz D.M., Dahlbäck B. (2013). Coagulation factor VA2440G causes east Texas bleeding disorder via TFPIα. J. Clin. Invest..

[11] Kuang S.Q., Hasham S., Phillips M.D., Wolf D., Wan Y., Thiagarajan P. (2001). Characterization of a novel autosomal dominant bleeding disorder in a large kindred from east Texas. Blood.

[12] Zimowski K.L., Petrillo T., Ho M.D., Wechsler J., Shields J.E., Denning G. (2021). F5-Atlanta: a novel mutation in F5 associated with enhanced east Texas splicing and FV-short production. J. Thromb. Haemost..

[13] Cunha M.L., Bakhtiari K., Peter J., Marquart J.A., Meijers J.C., Middeldorp S. (2015). A novel mutation in the F5 gene (factor V Amsterdam) associated with bleeding independent of factor V procoagulant function. Blood.

[14] Peterson J.A., Gupta S., Martinez N.D., Hardesty B., Maroney S.A., Mast A.E. (2022). Factor V east Texas variant causes bleeding in a three-generation family. J. Thromb. Haemost..

[15] Esmon C.T., Owen W.G. (1981). Identification of an endothelial cell cofactor for thrombin-catalyzed activation of protein C. Proc. Natl. Acad. Sci. U. S. A..

[16] Esmon C.T., Esmon N.L., Harris K.W. (1982). Complex formation between thrombin and thrombomodulin inhibits both thrombin-catalyzed fibrin formation and factor V activation. J. Biol. Chem..

[17] Esmon C. (2000). The protein C pathway. Crit. Care Med..

[18] Kalafatis M., Egan J.O., van't Veer C., Cawthern K.M., Mann K.G. (1997). The regulation of clotting factors. Crit. Rev. Eukaryothic Gene Expr..

[19] Mann K.G., Kalafatis M. (2002). Factor V: a combination of Dr. Jekyll and Mr. Hyde. Blood.

[20] Kalafatis M., Mann K.G. (1993). Role of the membrane in the inactivation of factor Va by activated protein C. J. Biol. Chem..

[21] Bakker H.M., Tans G., Claessen T.J., Thomassen M.C., Hemker H.C., Griffin J.H. (1992). The effect of phospholipids, calcium ions and protein S on rate constants of human factor Va inactivation by activated human protein C. Eur. J. Biochem..

[22] Mann K.G., Hockin M.F., Begin K.J., Kalafatis M. (1997). Activated protein C cleavage of factor Va leads to dissociation of the A2 domain. J. Biol. Chem..

[23] Kalafatis M., Rand M.D., Mann K.G. (1994). The mechanism of inactivation of human factor V and human factor Va by activated protein C. J. Biol. Chem..

[24] Nicolaes G.A., Tans G., Thomassen M.C.L., Hemker H.C., Pabinger I., Varadi K. (1995). Peptide bond cleavages and loss of functional activity during inactivation of factor Va and factor VaR506Q by activated protein C. J. Biol. Chem..

[25] Kalafatis M., Bertina R.M., Rand M.D., Mann K.G. (1995). Characterization of the molecular defect in factor V R506Q. J. Biol. Chem..

[26] Dahlbäck B. (1995). Resistance to activated protein C, the Arg506 to Gin mutation in the factor V gene, and venous thrombosis. Thromb. Haemost..

[27] Dahlback B. (1997). Resistance to activated protein C caused by the factor VR506Q mutation is a common risk factor for venous thrombosis. Thromb. Haemost..

[28] Odegaard B., Mann K. (1987). Proteolysis of factor Va by factor Xa and activated protein C. J. Biol. Chem..

[29] Barhoover M.A., Kalafatis M. (2011). Cleavage at both Arg306 and Arg506 is required and sufficient for timely and efficient inactivation of factor Va by activated protein C. Blood Coagul. Fibrinolysis.

[30] Thorelli E., Kaufman R.J., Dahlbӓck B.r. (1999). Cleavage of factor V at Arg 506 by activated protein C and the expression of anticoagulant activity of factor V. Blood.

[31] Yegneswaran S., Kojima Y., Nguyen P.M., Gale A.J., Heeb M.J., Griffin J.H. (2007). Factor Va residues 311-325 represent an activated protein C binding region. J. Biol. Chem..

[32] Aparicio C., Dahlbäck B. (1996). Molecular mechanisms of activated protein C resistance. Properties of factor V isolated from an individual with homozygosity for the Arg506 to Gln mutation in the factor V gene. Biochem. J..

[33] Norstrøm E.A., Steen M.r., Tran S., Dahlbäck B.r. (2003). Importance of protein S and phospholipid for activated protein C-mediated cleavages in factor Va. J. Biol. Chem..

[34] Dahlback B. (1997). Factor V and protein S as cofactors to activated protein C. Haematologica.

[35] Shen L., Dahlbäck B. (1994). Factor V and protein S as synergistic cofactors to activated protein C in degradation of factor VIIIa. J. Biol. Chem..

[36] Dahlbäck B., Hildebrand B. (1994). Inherited resistance to activated protein C is corrected by anticoagulant cofactor activity found to be a property of factor V. Proc. Natl. Acad. Sci. U. S. A..

[37] Kalafatis M., Haley P.E., Lu D., Bertina R.M., Long G.L., Mann K.G. (1996). Proteolytic events that regulate factor V activity in whole plasma from normal and activated protein C (APC)-resistant individuals during clotting: an insight into the APC-resistance assay. Blood.

[38] Gale A.J., Tsavaler A., Griffin J.H. (2002). Molecular characterization of an extended binding site for coagulation factor Va in the positive exosite of activated protein C. J. Biol. Chem..

[39] Segers K., Dahlbäck B., Rosing J., Nicolaes G.A. (2008). Identification of surface epitopes of human coagulation factor Va that are important for interaction with activated protein C and heparin. J. Biol. Chem..

[40] Ruben E.A., Rau M.J., Fitzpatrick J.A.J., Di Cera E. (2021). Cryo-EM structures of human coagulation factors V and Va. Blood.

[41] Steen M., Tran S., Autin L., Villoutreix B.O., Tholander A.L., Dahlback B. (2008). Mapping of the factor Xa binding site on factor Va by site-directed mutagenesis. J. Biol. Chem..

[42] Wood J.P., Bunce M.W., Maroney S.A., Tracy P.B., Camire R.M., Mast A.E. (2013). Tissue factor pathway inhibitor-alpha inhibits prothrombinase during the initiation of blood coagulation. Proc. Natl. Acad. Sci. U. S. A..

[43] Dahlbäck B., Guo L.J., Livaja-Koshiar R., Tran S. (2018). Factor V-short and protein S as synergistic tissue factor pathway inhibitor (TFPI α) cofactors. Res. Pract. Thromb. Haemost..

[44] Heeb M.J., Kojima Y., Hackeng T.M., Griffin J.H. (1996). Binding sites for blood coagulation factor Xa and protein S involving residues 493–506 in factor Va. Protein Sci..

[45] Kumar S., Stayrook S., Camire R.M., Krishnaswamy S. (2015). The X-ray structure of a variant of human factor V provides structural insights into the procofactor activation paradox. Blood.

[46] Kumar S., Stayrook S., Huntington J.A., Camire R.M., Krishnaswamy S. (2011). High resolution X-ray structure of snake venom factor V: evolution of a hemostatic cofactor to a toxin poised to inflict maximal damage to mammalian blood coagulation. Blood.

[47] Kumar S., Deng W., Stayrook S., Li R., Camire R.M., Krishnaswamy S. (2016). Structural basis for the procofactor to cofactor transition in human factor V. Blood.

[48] Thorelli E., Kaufman R.J., Dahlb„ck B. (1998). The C-terminal region of the factor V B-domain is crucial for the anticoagulant activity of factor V. J. Biol. Chem..

[49] Tracy P.B., Eide L.L., Bowie E.J.W., Mann K.G. (1982). Radioimmunoassay of factor V in human plasma and platelets. Blood.

[50] Camire R.M., Kalafatis M., Cushman M., Tracy R.P., Mann K.G., Tracy P.B. (1995). The mechanism of inactivation of human platelet factor Va from normal and activated protein C-resistant individuals. J. Biol. Chem..

[51] Gould W.R., Silveira J.R., Tracy P.B. (2004). Unique *in vivo* modifications of coagulation factor V produce a physically and functionally distinct platelet-derived cofactor: characterization of purified platelet-derived factor V/Va. J. Biol. Chem..

[52] Higgins D.L., Mann K.G. (1983). The interaction of bovine factor V and factor V-derived peptides with phospholipid vesicles. J. Biol. Chem..

[53] Katzmann J.A., Nesheim M.E., Hibbard L.S., Mann K.G. (1981). Isolation of functional human coagulation factor V by using a hybridoma antibody. Proc. Natl. Acad. Sci. U. S. A..

[54] Bradford H.N., Krishnaswamy S. (2019). Occlusion of anion-binding exosite 2 in meizothrombin explains its impaired ability to activate factor V. J. Biol. Chem..

[55] Kalafatis M., Krishnaswamy S., Rand M.D., Mann K.G. (1993). [13] Factor V. Methods Enzymol..

[56] Broze G.J., Lange G.W., Duffin K.L., MacPhail L. (1994). Heterogeneity of plasma tissue factor pathway inhibitor. Blood Coagul. Fibrinolysis.

[57] Betz A., Krishnaswamy S. (1998). Regions remote from the site of cleavage determine macromolecular substrate recognition by the prothrombinase complex. J. Biol. Chem..

[58] Eaton S.L., Hurtado M.L., Oldknow K.J., Graham L.C., Marchant T.W., Gillingwater T.H. (2014). A guide to modern quantitative fluorescent western blotting with troubleshooting strategies. J. Vis. Exp..

[59] Bradford H.N., Orcutt S.J., Krishnaswamy S. (2013). Membrane binding by prothrombin mediates its constrained presentation to prothrombinase for cleavage. J. Biol. Chem..

