# Supplementary material for: Regulation of factor V by the anticoagulant protease activated protein C: Influence of the B-domain and TFPIα
Source: J Biol Chem. 2022 Sep 30;298(11):102558. doi: 10.1016/j.jbc.2022.102558 (PMC9637641; doi:10.1016/j.jbc.2022.102558)
Supplement: Supplemental Fig. S1–S3 [file mmc1.docx]

**Regulation of Factor V by the Anticoagulant Protease Activated Protein C: Influence of the B-domain and TFPIα**

Francis Ayombil^1^, Teodolinda Petrillo^1^, Haein Kim^1^, Rodney M. Camire^1,2*^

^1^Division of Hematology and the Raymond G. Perelman Center for Cellular and Molecular Therapeutics, The Children’s Hospital of Philadelphia, Philadelphia PA 19104

^2^Department of Pediatrics, Perelman School of Medicine, University of Pennsylvania, Philadelphia, PA, 19104

Present address for Teodolinda Petrillo: The University of Maryland, Baltimore, MD 21201

Present address for Haein Kim: JDRF T1D Fund, 50 Milk Street, 16^th^ floor, Boston, MA 02109

*Corresponding author: Rodney M. Camire

E-mail: [rcamire@pennmedicine.upenn.edu](mailto:rcamire@pennmedicine.upenn.edu)

Running title: Regulation of FV inactivation by the B-domain

**Keywords:** factor V, acidic and basic residues, procofactor, tissue factor pathway inhibitor, factor Va, cofactor, prothrombinase, coagulation factor, thrombin, protein complex, hemostasis

**Supporting Information Figures**

**Figure S1**

**Figure S1 Proteolysis of FV-B152 is not impaired by FV-BR fragment.** Cleavage of 20 nM FV-B152 by 1.0 nM APC in the absence (*A)* or presence of 250 nM FV-BR *(B)* was monitored over time. Samples were resolved by SDS-PAGE and immunoblotted as in Fig. 2. In panel *C*, band density of starting material (*A & B*) was expressed as a function of time; FV-B152 (○), and FV-B152 + FV-BR (●). Data are representative of two similar experiments.

**Figure S2**

**Figure S2. Proteolysis of FV and FVa is not altered by exogeneous BR fragments.** Cleavage of PD-FV *(A),* or FVa *(B)* by APC were followed in the absence (●), and presence of TFPIα-BR (○) or FV-BR (■). Following quantitative densitometry, the data were expressed as a percentage of the band intensity of the starting material (n = 3; mean ± SD)%.

**Figure S3**

**Figure S3. BR fragments and TFPI⍺ do not alter APC activity *in vitro*.** Hydrolysis of the peptidyl substrate S-2366 (0.5 µM) was followed continuously over time in the absence (○), or presence of 1 nM APC (●) with FV-BR (△), TFPIα-BR (▽) and TFPI⍺ (□) at 250 nM. The data were each fit to a linear equation to obtain slopes (rate). These data are representative of two similar experiments.
